# Carcinosarcoma of the Esophagus—A Diagnostic Challenge

**DOI:** 10.31486/toj.23.0007

**Published:** 2023

**Authors:** Vishu Jain, Peeyush Varshney, Divya Aggarwal, Subhash Chandra Soni, Vaibhav Kumar Varshney, B Selvakumar, Lokesh Agarwal

**Affiliations:** ^1^ Department of Surgical Gastroenterology, All India Institute of Medical Sciences, Jodhpur, Rajasthan, India; ^2^ Department of Pathology and Laboratory Medicine, All India Institute of Medical Sciences, Jodhpur, Rajasthan, India

**Keywords:** *Carcinosarcoma*, *deglutition disorders*, *esophageal neoplasms*

## Abstract

**Background:** Esophageal carcinosarcoma is an uncommon histologic variant of esophageal malignancy, occurring in approximately 0.5% to 2.8% of patients. Esophageal carcinosarcoma usually involves the middle and lower esophagus and consists of both epithelial and mesenchymal components.

**Case Report:** A 54-year-old male presented with painless progressive dysphagia associated with loss of weight for 2 months. Esophagogastroduodenoscopy suggested an ulceroproliferative polypoidal growth in the lower thoracic esophagus. Biopsies from the growth showed leiomyosarcoma with tumor cells immunopositive for vimentin, h-Caldesmon, and smooth muscle actin and negative for pan-cytokeratin. Imaging suggested a heterogeneously enhancing polypoidal growth arising in the lower third of the esophagus. Thoracoscopic-assisted McKeown esophagectomy with gastric pull-up and standard 2-field lymphadenectomy was performed. A minor epithelial component was identified on final pathologic examination in addition to the leiomyosarcoma found on the preoperative biopsy. This epithelial component was invasive squamous cell carcinoma and was positive for pan-cytokeratin and p40, both of which were negative in the sarcomatous component. The patient received 4 cycles of adjuvant chemotherapy (carboplatin and paclitaxel). However, he developed a recurrence in the left cervical lymph node 4 months after adjuvant treatment and died 2 months after the diagnosis of recurrence.

**Conclusion:** Carcinosarcoma can be easily missed in the presence of predominantly sarcomatous components even on immunohistochemical analysis. These tumors may be associated with poor prognosis and may have early recurrence despite surgery and adjuvant treatment.

## INTRODUCTION

Esophageal malignancies are tenth in incidence of new cases of cancer, with 604,000 new cases diagnosed each year, and sixth in overall cancer mortality, with 544,000 deaths per year.^1^ Histologically, almost 90% of esophageal tumors are squamous cell carcinoma or adenocarcinoma.^2^ Carcinosarcoma is a rare histologic variant of esophageal malignancy, accounting for approximately 0.5% to 2.8% of all malignant tumors of the esophagus.^3,4^ In 1865, Virchow first proposed carcinosarcoma as a tumor with the proliferation of both epithelial and mesenchymal components.^5^ In 1904, Hansemann reported the first case of esophageal carcinosarcoma in the literature.^6^

We present the case of a 54-year-old male who was preoperatively diagnosed with sarcoma on endoscopic biopsy and underwent minimally invasive esophagectomy with standard 2-field lymphadenectomy. Final histopathology revealed carcinosarcoma. The patient received adjuvant chemotherapy but had an early recurrence.

## CASE REPORT

**Figure 1.**
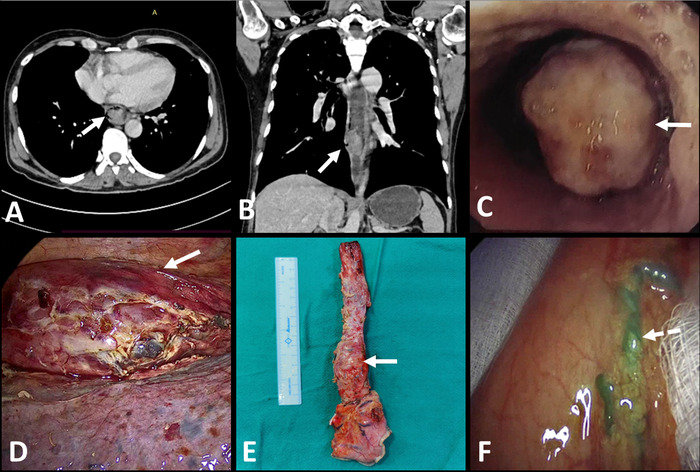
**(A) Axial and (B) coronal section of contrast-enhanced computed tomography of the thorax and abdomen showing polypoidal intraluminal growth. (C) Endoscopic image showing ulceroproliferative polypoidal intraluminal growth. (D) Intraoperative image of the esophagus with tumor after thoracoscopic mobilization. (E) Postoperative esophagectomy specimen, and (F) confirmation of gastric conduit viability using indocyanine green dye.** Note: Arrows in (A), (B), (C), (D), and (E) indicate tumor; dotted arrow in (F) indicates flow of indocyanine green in gastric conduit.

A 54-year-old male presented with painless progressive dysphagia—he was only able to tolerate semisolids (grade 3)—associated with weight loss for the prior 2 months. The patient's medical history was significant for pulmonary tuberculosis 6 years prior (for which he completed the entire course of antitubercular treatment) and HIV infection for the prior 1 year (for which he was on antiretroviral therapy) with a CD4 count of 374 cells/mm^3^. Esophagogastroduodenoscopy (EGD) was suggestive of multiple extensive ulcerations in the midesophagus and an ulceroproliferative polypoidal esophageal growth extending from 32 to 38 cm from the incisors (Figure 1). Multiple biopsies taken from the ulcers and the growth showed leiomyosarcoma with tumor cells immunopositive for vimentin, h-Caldesmon, and smooth muscle actin (SMA) but negative for pan-cytokeratin (pan-CK), p40, S100P, and CD117. A malignant epithelial component was not identified in the preoperative biopsy as no pan-CK–positive cells were identified.

Contrast-enhanced computed tomography (CT) of the neck, thorax, and abdomen showed a heterogeneously enhancing polypoidal growth arising from the posterior wall of the lower third of the esophagus measuring 6 × 4 cm without any prominent lymphadenopathy (Figure 1). Magnetic resonance imaging showed a well-defined heterogeneously enhancing polypoidal lesion arising from the posterior wall of the lower third of the thoracic esophagus that was hyperintense on T1/T2 images with diffusion restriction and measured 5.5 × 1.8 × 3.6 cm, causing significant narrowing of the lumen and suggesting leiomyosarcoma.

The patient underwent thoracoscopic-assisted McKeown esophagectomy with gastric pull-up and standard 2-field lymphadenectomy. The patient's postoperative course was uneventful, with strict adherence to perioperative enhanced recovery after surgery protocols.^7^

**Figure 2.**
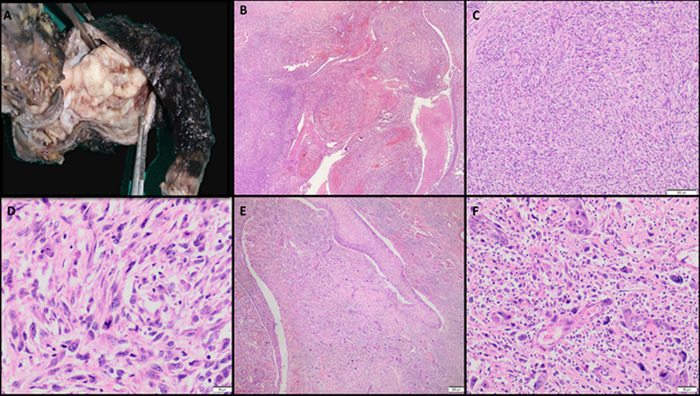
(A) Gross examination showed a polypoidal mass in the lumen. (B) Two tumors were admixed with a few infiltrative nests and many interlacing fascicles (×10 magnification). The sarcoma component had (C) interlacing fascicles of cells (hematoxylin and eosin, ×20 magnification) with cells showing (D) marked nuclear pleomorphism, coarse chromatin, prominent nucleoli and a moderate amount of cytoplasm with indistinct cell borders (×200 magnification). The carcinoma component had (E) infiltrating nests of cells (×20 magnification) with (F) squamoid differentiation (×200 magnification).

**Figure 3.**
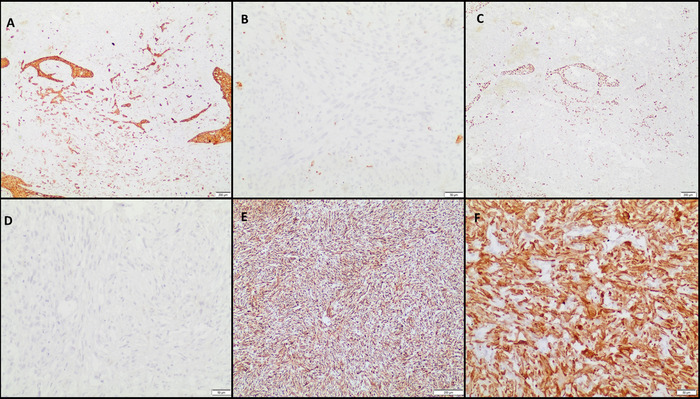
(A) The carcinoma component was positive for pan-cytokeratin (pan-CK) (immunohistochemistry [IHC], ×20 magnification) while (B) the sarcoma component was negative for pan-CK (IHC, ×200 magnification). (C) The carcinoma component was positive for p40 (IHC, ×20 magnification) but (D) the sarcoma component was negative for p40 (IHC, ×200 magnification). The sarcoma component was positive for (E) vimentin (IHC, ×100 magnification) and (F) smooth muscle actin (IHC, ×200 magnification).

Gross examination of the specimen revealed a polypoidal irregular lower esophageal growth measuring 4.8 × 3.0 × 3.0 cm with homogeneous grey-white firm cut surface (Figure 2). Microscopy revealed a minor epithelial component in addition to the leiomyosarcoma found on preoperative biopsy. The epithelial component was invasive squamous cell carcinoma and was positive for pan-CK and p40, both of which were negative in the sarcomatous component (Figure 3). The immunoprofile of the sarcomatous component was similar to that of the preoperative biopsy. Some areas of chondroid differentiation were also noted in the final biopsy. Because the tumor had reached the muscularis propria and invaded it but had not reached the adventitia, the pathologic stage was pT2N0 as per the 8th edition of the American Joint Committee on Cancer staging manual,^8^ with all margins free. The tumor was classified as stage II.

A multidisciplinary tumor board recommended adjuvant chemotherapy. The patient received 4 cycles of adjuvant chemotherapy (carboplatin and paclitaxel). However, he developed a tumor recurrence in the left cervical lymph node with clinically palpable neck swelling 4 months after completion of chemotherapy. Positron emission tomography with 2-deoxy-2-[fluorine-18]fluoro-D-glucose integrated with CT revealed metabolically active multiple left cervical, left supraclavicular, mediastinal, left internal mammary and perigastric lymph nodes; a subcapsular liver lesion; and malignant pleural effusion. Palliative chemotherapy was planned for the patient, but his family opted for supportive treatment, and the patient died 2 months after the diagnosis of recurrence.

## DISCUSSION

The origin of the sarcomatous component in carcinosarcoma is unclear and has led to various names, including spindle cell carcinoma, pseudosarcomatous squamous cell carcinoma, polypoid carcinoma, and squamous cell carcinoma with a spindle cell component.^9^ One theory suggests that sarcomatous components are sarcoma-like cells originating from squamous epithelium.^10^ Another theory suggests carcinosarcoma to be a concurrent sarcoma because of the cytologic pleomorphism and the high mitotic rate of these cells.^11^ A third theory describes a pseudosarcomatous component originating from mesenchymal metaplasia of squamous cells.^12^ Multiple studies based on electron microscopy and immunohistochemistry suggest some degree of epithelial differentiation in sarcoma components.^13,14^ Zinc finger E-box-binding homeobox 1, which has been associated with epithelial-mesenchymal transition transcription factors, is expressed in the sarcoma component of esophageal carcinosarcoma, suggesting that esophageal carcinosarcoma originates from the epithelial-mesenchymal transition phenomenon.^15,16^

Carcinosarcoma usually occurs in middle-aged and elderly patients with a predilection for the middle and lower third of the esophagus (approximately 80%) because of the presence of smooth muscle in that area.^9,17^ The carcinosarcoma primarily appears as an intraluminal growth rather than as esophageal wall thickening.^9^ The sarcomatoid component can have osseus, chondroid, or skeletal muscle differentiation, and our patient also had some areas of chondroid differentiation. On immunohistochemistry, the presence of 2 different tumor components needs to be established with a panel of antibodies, as prognosis is usually guided by the epithelial component, which is more aggressive than the sarcomatoid component. Carcinoma markers include pan-CK and epithelial membrane antigen; p40 or p63 can aid with confirmation of squamoid differentiation. The sarcoma component should be negative for these markers and can be confirmed with vimentin. Further workup of the sarcomatous component depends on the morphologic differentials and may include a panel of antibodies including SMA, desmin, h-Caldesmon, S100P, SOX10, CD34, and myogenin. Sarcomatoid squamous cell carcinoma is a principal differential diagnosis in these cases, and combining the above markers helps differentiate sarcomatoid squamous cell carcinoma from carcinosarcoma. While the sarcomatoid component of sarcomatoid squamous cell carcinoma shows positivity for both pan-CK and p40, these markers are not expressed in the sarcomatous component of carcinosarcoma.

The presence of 2 types of morphology and an immunopositive signature in a single entity create a particular challenge for the pathologist. A minor epithelial or sarcomatous component may not be sampled in a preoperative biopsy, as in our case. No epithelial component could be identified in the biopsy even on retrospective review. The preoperative biopsy is like the tip of the iceberg and may not represent the actual entity. The preoperative diagnosis can vary depending on the site of the biopsy, the proportion of each component in the tumor, and the number of cores taken. Detailed gross examination and thorough tumor sampling are essential for reaching the correct diagnosis in the resection specimen, as a minor epithelial component is easy to miss in such cases.

Clinically, carcinosarcomas have a similar presentation—dysphagia and/or chest pain—as other esophageal malignancies. Patients are then investigated with an EGD, tissue biopsy, and contrast-enhanced CT. Immunohistochemistry of the biopsy tissue establishes the preoperative diagnosis. Carcinosarcomas have a low incidence; hence, no management guidelines have been established. Patients are treated for esophageal malignancies and traditionally planned for upfront resection without distant metastasis.^4,18^ Limited literature is available on the role of adjuvant treatment and chemotherapy regimens, including DCF (docetaxel, cisplatin, and 5-fluorouracil) and DP (docetaxel + cisplatin).^4^

In our case, we performed a minimally invasive esophagectomy with standard 2-field lymphadenectomy done routinely at our center for esophageal carcinoma. The patient's postoperative course was uneventful, and he was discharged on postoperative day 5. However, the patient had an early recurrence despite R0 resection and adjuvant chemotherapy. In a retrospective study of 28 patients who underwent esophagectomy, Hashimoto et al reported 3- and 5-year recurrence-free survival rates of 66.4% and 61.6%, respectively, and 3- and 5-year overall survival rates of 73% and 61.9%, respectively.^18^ Most patients with esophageal carcinosarcoma (54.5% to 95.8%) are detected in the early stage because the polypoidal nature of the tumor causes early symptoms.^4^ The incidence of invasion to adjacent organs in esophageal carcinosarcoma is also lower than that of squamous cell carcinoma, so carcinosarcomas are more resectable than squamous cell carcinoma even at an advanced stage. Thus carcinosarcoma is associated with a better prognosis for esophageal carcinosarcoma compared to squamous cell carcinoma.^4^ However, our patient had an aggressive course with early recurrence in the form of distant lung and liver metastases within 4 months of resection. One possible reason for early recurrence in our patient could be his immunocompromised status as a result of retroviral disease, which could explain the aggressive behavior of the tumor.^19^ The presence of a sarcoma component might explain the hematogenous metastases of the tumor to the lung and liver.^20^

## CONCLUSION

Carcinosarcoma is a rare malignant neoplasm of the esophagus. Proper histopathologic analysis, including immunohistochemistry, is critical as epithelial components can be easily missed in the presence of predominantly sarcomatous components. Minimally invasive esophagectomy is safe and feasible in these patients. However, carcinosarcomas can be associated with poor prognosis and early recurrence despite adequate surgery and adjuvant treatment. Further reports and studies are needed to formulate the optimal guidelines for the management of carcinosarcoma.

## Notes

*This article meets the Accreditation Council for Graduate Medical Education and the American Board of Medical Specialties Maintenance of Certification competencies for Patient Care and Medical Knowledge.*
